# Cleft Lip – A Comprehensive Review

**DOI:** 10.3389/fped.2013.00053

**Published:** 2013-12-27

**Authors:** Mahdi A. Shkoukani, Michael Chen, Angela Vong

**Affiliations:** ^1^Department of Otolaryngology, Wayne State University School of Medicine, Detroit, MI, USA

**Keywords:** xcleft lip, orofacial clefting, nasal deformity, cleft lip repair, congenital abnormalities

## Abstract

Orofacial clefts comprise a range of congenital deformities and are the most common head and neck congenital malformation. Clefting has significant psychological and socio- economic effects on patient quality of life and require a multidisciplinary team approach for management. The complex interplay between genetic and environmental factors play a significant role in the incidence and cause of clefting. In this review, the embryology, classification, epidemiology, and etiology of cleft lip are discussed. The primary goals of surgical repair are to restore normal function, speech development, and facial esthetics. Different techniques are employed based on surgeon expertise and the unique patient presentations. Pre-surgical orthopedics are frequently employed prior to definitive repair to improve outcomes. Long term follow up and quality of life studies are discussed.

## Introduction

Orofacial clefts include a range of congenital deformities most commonly presenting as cleft lip with or without cleft palate (CLP) or isolated cleft palate (CP). CLP is the second most common congenital birth defect in the U.S. trailing only Down syndrome (1). There are roughly 7,000 infants born with orofacial clefts in the U.S. annually (1). Beyond the physical effects on the patient, CLP also has significant psychological and socioeconomic effects on both patient and family, including disruption of psychosocial functioning and decreased quality of life (QOL) (2, 3). It is associated with increased mortality from many causes, including suicide (4) as well as substantial healthcare costs (5). Cleft lips can be unilateral or bilateral, and may involve the alveolus or palate. Affected individuals may present with other congenital anomalies and may be part of a genetic syndrome.

Efforts are ongoing to uncover the epidemiology and etiology of this condition. The WHO-supported international collaborative research on craniofacial anomalies project establishes a global network to compile a comprehensive database and coordinate research strategies (6). Optimal management of a child with cleft lip demands an organized multidisciplinary effort involving the fields of otolaryngology, plastic surgery, maxillofacial surgery, orthodontistry, speech therapy, pediatrics, nursing, genetics counseling, audiology, psychology, and social work (7). The goals are to optimize feeding, facial growth, and speech and language development. One of the primary roles of the otolaryngologist is surgical repair to restore normal feeding, speech, and appearance. Successful repair of the cleft lip is simultaneously rewarding and challenging.

## Embryology

The embryological development of the lip and palate is well documented. Normal lip development occurs between weeks 4 and 8 of gestation. By the end of week 4, the frontonasal prominence forms from migrating neural crest cells of the first pharyngeal arch. Nasal placodes, representing ectodermal thickening, develop at the caudal end of this structure and divide the paired medial and lateral nasal processes. The primary palate forms from the fusion of the paired medial nasal processes by week 6, giving rise to the premaxilla: central upper lip, maxillary alveolar arch and four incisor teeth, and hard palate anterior to the incisive foramen (8, 9).

The secondary palate develops after the primary palate during weeks 6–12. The medial projections of the maxillary processes form palatal shelves which rise above the tongue, fusing medially at the midline, anteriorly with the primary palate, and superiorly with the septum. The incisive foramen marks the anterior extent of the secondary palate. Formation of the primary and secondary palates completes the separation of nasal and oral cavities, permitting simultaneous respiration, and mastication (10).

Normal development occurs sequentially, thus cleft lip may or may not be associated with cleft palate. Similarly, isolated cleft palate may arise independently of cleft lip. Deformities of the lip, palate, and nose are a result of the disruption of normal development. The severity is dictated by the timing, severity, and amount of disruption (11). A critical period is immediately before the formation of the primary palate and central lip, as the lateral nasal process undergoes a burst of mitotic growth. During this period, development is highly vulnerable to genetic and teratogenic effects (7).

## Classification

**Figure 1:**
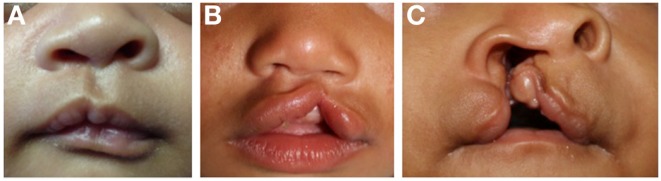
**Unilateral cleft lip**. **(A)** Microform type, **(B)** incomplete type, **(C)** complete type.

CLP is traditionally classified by phenotype, which can have variable expression ranging from microform to complete clefting, and may involve the alveolar ridge and palate (Figure 1). Phenotypes have been correlated with specific genetic linkage patterns, suggesting a possible correlation. CLP and CP are embryologically distinct processes from disruption at different stages of development and possess unique epidemiologic and genetic features (7, 10, 12).

CLP refers to a clinical spectrum of cleft lip with or without associated cleft palate (Table 1). Palate involvement generally denotes a related but more severe form of this anomaly, although they may have epidemiologic differences (12). Lip clefting may be complete (involving the full vertical height of the lip) or incomplete. Complete cleft lips are often associated with an alveolar cleft. The soft tissue bridge spanning the cutaneous lip or alveolus in an incomplete cleft lip is termed Simonart’s band and consists primarily of skin with variable amounts of orbicularis oris muscle fibers (13).

**Table 1:** **Anatomy of the cleft lip**.

	Normal	Unilateral CL	Bilateral CL
**Skin**	Intact across lip	Deficient across full (complete) or partial (incomplete) vertical height of upper lip	Deficient across full (complete) or partial (incomplete) vertical height of upper lip
**Muscle** (orbicularis oris)	Intact across lip	Usually deficient and/or disoriented across cleft	Usually deficient and/or disoriented across cleft
	Circumferentially orientated	Inserts along cleft or nasal base	Absent in prolabium
**Lip**	Cupid’s bow and philtrum present and symmetrical	Cupid’s bow is less conspicuous and upwardly rotated toward the cleft side. Philtral column is shorter on the cleft side	Bilateral loss of Cupid’s bow and philtral structures
**Bone** (premaxilla)	Intact	Depending on the involvement of alveolus, it may range from intact to a wide alveolar cleft	May be significantly protruded
**Nose**	Normal/symmetric nasal tip Normal/symmetric columella Normal/symmetric nasal base Nostril oriented vertically Normal caudal septum	Nasal tip flat and deflected to non-cleft side Short columella on cleft side Lateral crus of alar cartilage is displaced laterally, posteriorly, and inferiorly on cleft side Nostril oriented horizontally on cleft side Caudal septum is displaced to non-cleft side	Nasal tip flat and broad in bilateral complete cases only otherwise it Short columella Bilateral lateral crura of alar cartilages are displaced laterally, posteriorly, and inferiorly Nostril is oriented horizontally on both sides

Unilateral cleft lip (Figure 1) is associated with typical deformities caused by asymmetric forces on the premaxilla during facial growth. The presence of Simonart’s band may reduce the extent of facial deformity with growth by exerting a restorative force (14). There is rotation and distortion of the vermillion with loss of Cupid’s bow and philtral landmarks on the cleft side. Orbicularis oris muscle fibers are asymmetrically oriented along the cleft margins and may be continuous across Simonart’s band in milder forms (15). Histologic studies have shown that the degree of disorientation of muscle fibers near the cleft correlate with cleft severity (16). Muscle volume does not appear to be reduced in the non-cleft portions of the lip (17). The typical nasal deformity is displacement of the ipsilateral lateral crus of the alar cartilage laterally, inferiorly, and posteriorly. The tip is flattened and deflected to the non-cleft side. The ipsilateral nostril is oriented horizontally rather than vertically. The columella is significantly shortened and deviates to the non-cleft side along with the caudal septum. The nasal cartilages may or may not be deficient (13).

**Figure 2:**
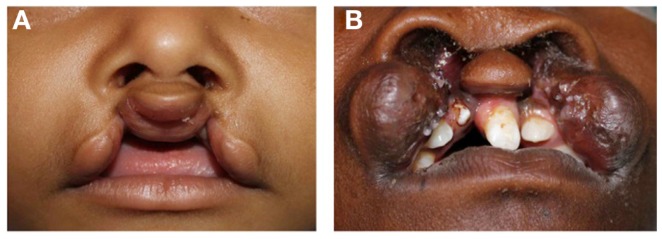
**Bilateral cleft lip**. **(A)** Incomplete type, **(B)** complete type.

In bilateral cleft lip, the premaxilla grows independently of the maxillae on either side and may protrude considerably, particularly in complete clefts (Figure 2) (18). The prolabium, consisting of soft tissues of the premaxilla without muscle fibers, also lacks Cupid’s bow and philtral columns bilaterally. The columella is severely shortened or absent while the lateral crura are displaced laterally, producing a broad, flat nasal tip.

Subclinical phenotypes likely lie within the extended spectrum of non-syndromic CLP. Examples include lip anomalies (19), dental anomalies, and facial morphometric features. Perhaps the best studied are orbicularis oris muscle defects in the absence of a visible cleft. These are assessed by high-resolution ultrasound (20, 21) and seem to preferentially occur in immediate relatives of those with cleft lip (19, 22). Identification of subclinical phenotypes may expand the search for susceptible genes.

In contrast to CLP, cleft palate (CP) is primarily characterized by disorientation of palatal muscles which lead to feeding difficulties, velopharyngeal insufficiency, and speech problems. The spectrum ranges from a submucosal cleft to complete clefting of the primary and secondary palate. They are more likely to be syndromic compared to CLP.

## Epidemiology

CLP occurs in 1 in 500–2,500 live births depending on ancestry, geographic location, maternal age, prenatal exposures, and socioeconomic status (2, 7). The latest CDC estimates report the incidence of CLP to be 1 in 940 live births, with 4,437 cases every year (1). More than 60% of orofacial clefts involve the lip (23). It was reported that isolated cleft lips alone accounts for about 10–30%; combined primary and secondary palate involvement comprises 35–55% of cases; involvement of secondary palate alone accounts for 30–45% of cases (24).

Major population differences have been reported, with the highest rates in Asians and Native Americans (1 in 500 births) and the lowest rate in Africans (1 in 2,500 births) (25).

Cleft lip is consistently more common in males at a 2:1 ratio, in contrast to cleft palate which has a similar ratio in favor of females. Some have postulated that common maternal hormones may be involved in both sex determination and orofacial clefting (26).

Unilateral cleft lip shows a 2:1 left predominance (10, 27). While the mechanism is unclear, the observation that the facial artery develops slower on the left may be a factor (28). An association between cleft laterality and handedness has been proposed (29) but this has not been consistently shown (27, 30).

## Etiology

Epidemiologic and etiologic features of CLP differ in the syndromic and non-syndromic forms. Non-syndromic forms are the best studied and occur in 70% of cases (31, 32). The causes are multifactorial and involve genetics, environmental factors, and teratogens (Table 2) (10, 33).

**Table 2:** **Reported etiologies of non-syndromic cleft lip with or without cleft palate**.

**Genetics**
*IRF6*
*ch8q24*
*VAX1*
*FGFR2*
*BMP4*
**Maternal risk factors**
Smoking
Alcoholism
Pregestational diabetes
Gestational diabetes
Age >40 years
Folate deficiency
Zinc deficiency
**Teratogens**
Valproic acid
Phenytoin
Retinoic acid
Chemical solvents
Pesticides
Occupation-related (leather, shoemaking, healthcare)

Genetic susceptibility has long been identified as a major component of CLP. Monozygotic twin studies suggest that genetics account for 40–60% of orofacial clefting (34). However, the identification of candidate genes is complicated by heterogeneity, non-Mendelian inheritance patterns, and limited sample sizes (10). The interferon regulatory factor 6 (*IRF6*) gene is consistently associated with non-syndromic CLP in multiple studies (10, 35) and is also the causative agent of van der Woude syndrome, the most common syndromic cause of cleft lip. Recent availability of genome-wide association studies (GWAS) has identified several new genetic loci, including a “gene desert” region on chromosome 8q24 (36). The GENEVA Cleft Consortium study identified different distributions of IRF6 and ch8q24 genes in Europe and Asia, suggesting that associations may be population-specific (37). The list of candidate genes is long and includes VAX1 (37), FGFR2 (38), and BMP4 (39) among others.

Many environmental factors have been investigated in epidemiologic studies. Maternal smoking increases the risk of CLP by up to 30% (40) and a dose-response effect has been consistently reported (41, 42). Secondhand smoke exposure does not seem to increase risk (43). Maternal alcohol consumption is controversial, although binge drinking may increase risk (44). Confounding between cigarette and alcohol use occurs frequently and their effects should be analyzed independently. Pregestational diabetes, and to a lesser extent, gestational diabetes have been linked to non-cardiac defects including orofacial clefts (45). A recent meta-analysis showed that maternal age >40 years increased risk of CLP by 56% compared to maternal age between 20 and 29 years (46). Folate supplementation in early pregnancy has been found to reduce risk by one third (47) to three quarters (48), although not all studies have reported statistical significance (49). The protective effect varies between populations and this may reflect a strong genetic component in the folate metabolism pathway (50). Deficiency of zinc, an important element of neuronal migration, causes clefting in animals (51) and may increase risk in humans (52). In general, daily intake of multivitamins is recommended for all pregnant women due to potential benefits and minimal risks if taken properly (53).

Potential teratogens that have been reported include retinoic acid, phenytoin, and valproic acid (54). Other proposed risk factors include various occupational and chemical exposures, hyperthermia, stress, maternal obesity, oral hormone supplementation, ionizing radiation, and maternal infection (10, 53). The complex interplay between genetic and environmental factors undoubtedly play a role in the pathogenesis of CLP. Investigation of these interactions may open new avenues of research for prevention and management of CLP.

Thirty percent of newborns with CLP have additional congenital anomalies occurring as part of a syndrome (31, 32). Over 500 Mendelian syndromes are listed in the Online Mendelian Inheritance in Man (OMIM) database. The most common and well-known syndrome associated with cleft lip is van der Woude syndrome. It is caused by a defect in the *IRF6 gene* on chromosome 1 and is inherited in an autosomal dominant fashion. Typical clinical features are cleft lip and/or palate, lower lip pit or fistula, and dental anomalies (55). Popliteal pterygium syndrome is a similar syndrome with orofacial clefting, lower lip pits, popliteal webs, and genitourinary anomalies. Other syndromes linked to CLP include Stickler syndrome, Hardikar syndrome, Treacher-Collins syndrome, siderius X-linked mental retardation, Loeys–Dietz syndrome, and Malpuech facial clefting syndrome (56).

## Goals of Surgical Repair

Cleft lip repair is complicated by the distortion of multiple anatomical structures, which can occur with varying severity. The challenges of reconstruction can be as distinct as the patient presentations of clefts: unilateral versus bilateral, narrow clefts versus wide clefts, syndromic patients versus non-syndromic patients as previously described. Each patient presents a new challenge to the surgeon attempting to repair the cleft. Yet, the goal of surgery remains the same: addressing the functional and cosmetic deformity of cleft lip. In order to achieve such goal, the repair should include the creation of an intact and appropriately sized upper lip to compensate for the loss of philtral height on the cleft side, repair of the underlying muscular structure for normal oral competence and function, and primary repair of nasal deformity (57).

## Timing of Surgery

What determines the optimal timing of surgical repair can vary based on surgeon preference, anesthetic risks, comorbid congenital anomalies, and perceived psychological impact on the family. Most surgeons repair the cleft lip around 10–12 weeks of age. The rule of 10’s is still applicable. It was recommended by Wilhelmsen and Musgrave that that repair of cleft lip should take place when the patient reaches the following cut-offs: weight 10 lbs, hemoglobin 10 g/dL, and white blood cell count <10,000 mm^3^ (58). It was Mallard who proposed the commonly used “rule of order 10” for the timing of repair stated as weight over 10 lbs, hemoglobin over 10 g, age over 10 weeks (59).

Recently, there had been published results that have shown some successful repairs in neonates 1–8 day old using the modified Tennison technique (60). Proponents of early intervention argue that while there is clear evidence of greater anesthetic complications for children under 12 months of age, there is limited evidence showing greater anesthetic risk for neonates as compared to surgery at 3 months of age. It is also proposed that the parents will gain a psychological benefit as their child will have the surgery and look “normal” when they return home. Granted that the child is otherwise healthy, several series have shown that esthetic and anesthetic results are comparable between repair in the neonatal period and repair at a much older age (60, 61). There are, however, drawbacks for performing the repair at an early stage. Many patients, especially in cases of wide clefts, will also require pre-surgical orthopedics for varying amounts of time to prepare the patient for definitive surgery (62).

## Pre-Surgical Considerations

Preliminary maneuvers can be used to aid in obtaining a satisfactory result during definitive surgical repair may be considered. These steps may especially be beneficial for patients with wide clefts with significant misalignment of the alveolar arches that may prevent proper alignment at the time of definitive surgery (62–64).

Lip adhesion is a preliminary procedure prior to definitive surgical repair to convert a complete cleft lip into an incomplete cleft so that the final lip repair may be completed with reduced tension. Typically performed within the first month of life, laterally based flaps obtained from the lip itself (65) or inferiorly based flaps of the vermillion (66) may be used. There are no universally accepted indications for the procedure. The lip adhesion procedure is less favored in that it requires the patient to undergo an additional surgery, may have increased scar formation, or may not correctly align the maxillary segments, and may have the potential to dehisce at the surgical site (62). Lip adhesion cases ought typically be limited to patients in which maxillary segments are expanded without collapse, as the procedure will provide no benefit if the segments are medially collapsed. Alveolar expansion is usually preferred in such cases (62). In staged repair of severe incomplete or complete unilateral cleft lips, following the use of Latham device, one group found that pre-surgical lip adhesion provided increased thickness of the lateral orbicular oris muscle that aided in reconstructing the philtral ridge (67). Preoperative lip taping is also an option (68). It has been described as having a similar effect to lip adhesion procedure, though its approach is less invasive (69). Lip taping can be used in conjunction with other pre-surgical orthopedics.

The use of pre-surgical orthopedics may also be considered and is used as an adjunct for guiding surgical repair. Bilateral cleft lip presents with a protrusion of the premaxilla and deficient columella. The development of pre-surgical orthopedics saw great changes over the past 50 years, and signifies the importance of a collaborative multidisciplinary team approach to the treatment of clefts. Surgeons in the nineteenth century initially excised the premaxillary segment, which unfortunately lead to development of mandibular pseudoprognathism, malocclusion, maxillary growth restriction, and midface deficiency (62). It subsequently became important to preserve and retract rather than excise the premaxilla so that the cleft lip repair may have the best esthetic result. Pre-surgical orthopedics refers to all techniques prior to the cleft lip repair. Such techniques can include parental finger massage of the prolabium, tape pressure on the labial segments, intraoral device fixation, or nasoalveolar molding (NAM) (70).

Orthopedic devices can be generalized into several categories: active versus passive; intraoral versus extraoral; pre-surgical versus post-surgical (71). Active maxillary appliances move alveolar maxillary segments into approximation with controlled force. The Latham appliance (72) was designed to anchor on the non-cleft maxillary segment intraorally and actively reposition the lateral alveolar cleft segments and reduce the protruded maxilla (72). Placement of the device also requires a surgical procedure as it consists of a two-piece maxillary splint anchored by pins to the palate, an expansion screw to adjust the palatal segments, and a chain to retract the premaxillary segment. Due to treatment variations, timing differences, and lack of normative data, the controversy regarding the use of pre-surgical orthopedics remains and the paucity of definitive clinical studies prevent the development of proven guidelines (62). Despite this, the use of pre-surgical orthopedics remains widely used and many centers continue to promote the improved surgical and esthetic results from its use.

Passive appliances, called NAM, deliver no force and gradually mold the alveolar segments and position the direction of growth via alveolar molding plates of acrylic. Newborn cartilage is believed to be soft and lacking in elasticity, and thus would make passive molding an easier endeavor (73). Initial devices were designed to correct alveolar cleft only (74, 75), and did not sufficiently address the nasal deformity that is associated with cleft lip. In unilateral cleft lip, the lower lateral nasal cartilage is typically found to be positioned laterally and inferiorly, leading to a depressed dome, increased alar rim, oblique columella, and overhanging nasal apex (76). In bilateral cleft lip, the associated nasal deformity is a widened alar base and flattened nasal tip with a nearly absent columella. NAM devices reduce the alveolar gap and also include tape to bring the lip segments together and lengthen the columella, and nasal stents to help correct the nasal deformity (75). Additionally, NAM contributes to improved definitive surgical repair and helps reduce the overall number of surgical procedures to correct the facial deformity (77). Injection of botulinum toxin into the orbicularis oris muscle preoperatively has recently been suggested as a means of reducing tension and thus improve outcomes in cleft lip repair (77).

## Definitive Surgical Repair

### Unilateral cleft lip repair

**Figure 3:**
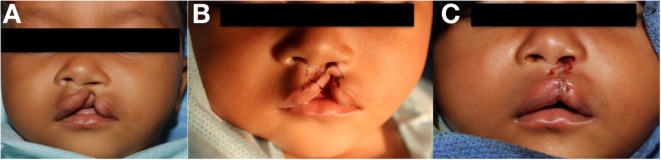
**Repair of left incomplete lip using triangular flap technique**. **(A)** Preoperative photo, **(B)** preoperative photo with marking, **(C)** status post repair.

Numerous techniques, as well as modifications to popular techniques, have been extensively described in the literature. Straight-line closure, or the Rose-Thompson closure, is an early technique introduced in the early twentieth century that may be procedure of choice for microform clefts (78), and is rarely used as primary technique for cleft repair. Straight-line closure typically resulted in notching of the lip and vertical scar contracture (79). Modern repair techniques have utilized the lateral lip flap to fill in the medial defect from the clefting of the lip. This concept, initially developed by Mirault, forms the basis for the modern rotation-advancement flaps, and the interdigitation of the triangular (Figure 3) (80, 81) or quadrangular flaps (82) to correct the cleft lip deformity (79). The triangular flap technique is a repair utilizing the interdigitation of triangular flaps. The concept underlying the technique can be similarly compared to a Z-plasty reconstruction of the lip. The advantage of this technique is that it is based on careful measurements based on landmarks and thus has less room for surgical judgment and flexibility at time of surgery. It may be more accommodating for wide clefts as compared to the rotation-advancement flap. The technique also preserves the Cupid’s bow. Limitations to the technique include the scar that is formed across the philtrum and the lack of flexibility (62, 79) as previously noted, no two clefts are the same and some measure of variability may often be required in achieving the intended esthetic result.

**Figure 4:**
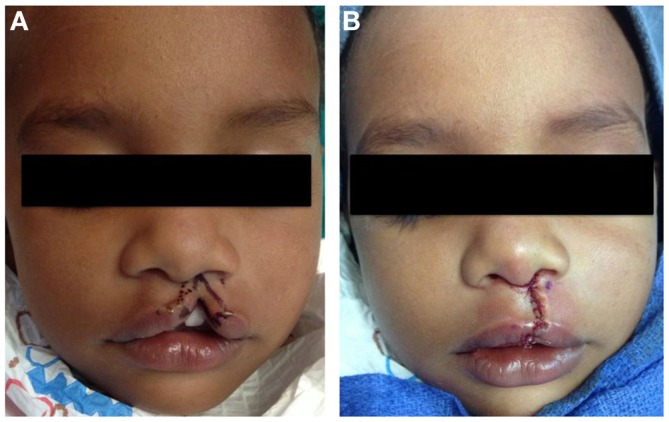
**Repair of left incomplete lip using modified Millard technique**. **(A)** Preoperative photo, **(B)** status post repair.

Millard’s rotation-advancement flap is currently the most prevalently used technique in cleft lip repair (Figure 4). Recent surveys have noted that 84% of response from practicing surgeons perform rotation advancement for complete unilateral cleft lip repair compared to 9% utilizing a triangular flap (83). The technique utilizes downward rotation of the superiorly displaced medial lip segment with advancement of the lateral lip flap to correct the defect below the nose (18, 59, 84). The many advantages of the technique belies its popularity, as it produces minimal tissue loss, creates a suture line consistent with the philtrum on the side of the cleft, preserves the Cupid’s bow, repositions the base of the nasal ala and provides tension to reduce nasal flare, guides the construction of a nasal sill, and is extremely versatile for the type of cleft the surgeon may come upon. The versatility of the technique also relies heavily on the surgeon expertise and experience, as it allows a great amount of surgical judgment in order to achieve a good esthetic result (62). The rotation-advancement flap relies on extensive undermining of the soft tissue of the maxilla in order to close wide clefts, especially in cases without prior lip adhesion so that the technique may not be ideal for wide clefts. In addition, the technique may occasionally sacrifice mucosa and lip tissue if the lateral lip segment has shorter vertical height. Thus, the rotation-advancement flap does not contribute well to the ideal construction of the lip (62). Crucial to the repair of the cleft lip is the reconstruction of the orbicularis oris muscle across the cleft. Proper alignment of the orbicularis muscle will allow for appropriate sphincter function (57). In order to accomplish this step, the orbicularis oris muscle must be freed from its attachments and aligned. The rotation-advancement flap is the most widely used, though not without modifications. Surveys of practicing physician have revealed that 45% will use a modified version of the rotation-advancement flap, with the most common being the Noordhoff vermilion flap, the Mohler modification, and the Onizuka triangular advancement flap, which will not be described here (83). Table 3 summarize the advantages and disadvantages of the different techniques.

**Table 3:** **Surgical techniques in cleft lip repair**.

Type of repair	Advantages	Disadvantages
Straight-line closure	Appropriate for microform clefts Rarely used for incomplete and complete clefts	Vertical scar contracture Sacrifice of normal tissue Notching of the lip Blunting of cupid’s bow
Geometric flaps	Appropriate for inexperienced surgeons Preserves Cupid’s bow Amenable to wide clefts	Lack of flexibility Scar violates the philtral subunit
Rotation-advancement flap	Versatility Minimal tissue loss Scar is hidden as a new philtral column Creates tension to reduce nasal flare	Mastered by experienced surgeons Possible small nostril on cleft side Extensive undermining necessary Vertical scar contracture

### Bilateral cleft lip repair

**Figure 5:**
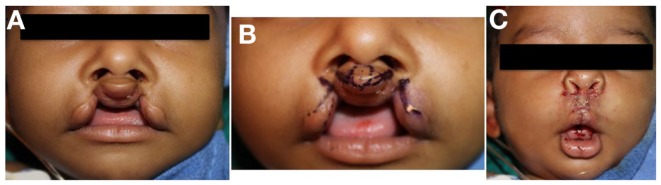
**Repair of bilateral incomplete lip**. **(A)** Preoperative photo, **(B)** preoperative photo with marking, **(C)** status post repair.

Repair of the bilateral cleft lip is often significantly more difficult than the unilateral cleft lip repair due to the presence of the premaxilla and prolabium. The prolabium is oftentimes deficient in muscle and vermillion, is small, and attached to the nasal tip with little or no columella present. The premaxilla also presents a challenge, as it remains highly variable in position and size; it can protrude forward and deviate to one side. The cleft maxillary alveolar arches are also often collapsed (62, 85). The surgeon is thus faced with the challenge of reconstructing the entire central portion of the lip with a deficient prolabium and abnormally displaced premaxilla. Initially, the premaxilla was resected to assist with closure. As mentioned above, this procedure produced an inferior cosmetic result. Attempts to approximate the lateral lip segments and suture to segments to the prolabium skin without re-establishing the muscle would also produce an inferior result. Mulliken (86) and Millard (87) advocated the re-establishment of orbicularis oris sphincter and addressing nasal deformity early on. Also, the width of alar base is reduced with these techniques. Figures shown demonstrate a commonly used technique popularized by Mulliken, and is described in detail elsewhere (86) (Figure 5). Complicated bilateral cleft repair would be an excellent example of the benefits of pre-surgical orthopedics or lip adhesion to aid in definitive surgical repair.

## Nasal Deformity

The nasal deformity associated with unilateral cleft lip is well defined since the 1950s (88). Due to fear of interfering with normal growth of the nose, surgeons rarely performed surgery to correct the nasal deformity until the 1970s (89). As previously described, in unilateral cleft lip, the lower lateral nasal cartilage is typically found to be positioned laterally and inferiorly, leading to a depressed dome, increased alar rim, oblique columella, and overhanging nasal apex (76). The goals of primary rhinoplasty include closure of the nasal floor, repositioning the lower lateral cartilages, and repositioning the alar base. The surgical repairs may be made through the same incisions that are used for the cleft lip repair, so that the nasal deformity can be addressed simultaneously (90, 91).

Within the past three decades, it is now commonplace for primary surgical correction of the nasal deformity at the time of definitive lip repair. It is now understood that the nasal growth on the cleft side of the nose is unaffected by early primary nasal surgery (89). This fact is supported by an 18-year follow up study comparing primary nasal repair with normal and unrepaired cleft lip children (92). The nasal deformity is of greater magnitude in bilateral cleft lips which includes bilateral alar deformity, widened alar base, and flattened nasal tip. The single stage repair involves preoperative Latham device followed by cleft lip and nasal repair. The surgical correction involves rim incisions with interdomal stitches between lower lateral cartilages and narrowing of the interalar distance. Excess skin is removed from the soft tissue triangle (86). Anthropometric analysis approximately 5 years post operatively found that nasal length, tip protrusion, and columellar width to be near normal values with symmetry of the lip and nose that were well preserved, validating the strength of the Mulliken procedure (93) in bilateral cleft lip and nasal deformity repair.

## Follow up

Facial growth in patients with CLP is often abnormal secondary to deformity and/or the surgical manipulations performed in an attempt to correct the deformity. In studies analyzing the growth of facial skeletons following cleft lip and/or palate repair, it can be collectively argued that the surgical manipulations performed in an attempt to correct the cleft can have greater effect on facial growth than the original deformity itself (94). In unoperated adult cleft patients, there remains normal potential for maxillary growth (94, 95). In isolated cleft lip, maxillary growth patterns following surgical repair is less clear. Animal studies have shown statistically significant effect on the cephalometric growth of the maxilla (96, 97). Clinical follow up, however, mostly fails to demonstrate appreciable effects on maxillary growth in children (95, 98). One noted report demonstrated functional closure of the lip significantly narrowed the transverse anterior cleft areas in early maxillary growth in patients with complete unilateral cleft lip and palate (99).

Long term follow up for patients following cleft repair is extremely important. As it stands, esthetic results from definitive surgical repair only come to reveal itself after some time has passed. Therefore it may be necessary for the patient to return to the operating room for revisions to improve function and appearance of the repair (85). Deformities following the initial surgeries can range from scars of the mucocutaneous, vermillion, or muscular regions (100). Recent changes in practices have led to some surgeons to advocate the use of modern medical adhesives as an adjunct technique for skin closure (101, 102). The same studies have shown that adhesives, like Dermabond, have offers equivalent mature wound cosmesis as traditional suture closure in the repair of cleft lip, and have the added benefit of avoiding additional dressing changes or suture removal under sedation (101, 102).

## Complications

Early mortality in the first few days of life of cleft lip patients, estimated to be 10–15% from reported literature (103, 104) is attributed to the lack of a “shake-down cruise” in which pediatricians have not yet had sufficient time to accurately assess and diagnose the patient with other congenital anomalies (58). However, current physicians tend toward the 10–10–10 rule, or in special cases, have the patient undergo pre-surgical orthopedics thereby delaying surgical intervention until the patient is older. Thus, complications related to comorbid congenital abnormalities or anesthetic complications are more easily avoided. As with any surgical procedures, there may be complications involved. Early studies have noted major complications for primary lip repair only are postoperative hemorrhage, breakdown of lip repair, pneumonia (4.3%), with minor complications as diarrhea, otitis media, partial separation of suture line, mild upper respiratory infection (58). Later studies have noted complications related to bleeding, feeding difficulty, wound dehiscence, wound infection, pneumonia, respiratory compromise, and respiratory arrest (105–107).

Historically, patients undergoing cleft lip repair have had post op inpatient hospitalization for monitoring (108). However, due to economic forces, changes in health care delivery, and the desire to return patients to a familiar home environment (109, 110), the trend has been toward same day discharge, necessitating reevaluation of postoperative complications. One study noted that emergent complications, if they occurred at all, would arise within 48 h and be due to respiratory difficulties (106). Notably, the four of the seven patients had known history of respiratory issues and were thus more susceptible to complications (106). Retrospective studies examining postoperative readmission and complication rates related to cleft lip repair in same day discharge patients suggests that carefully selected patients with no comorbid conditions and adequate home care will be unlikely to benefit from post op hospital observation as reasons for readmission were unrelated to surgical procedure, related to other comorbid conditions, and/or occurred well beyond the 1- to 2-day observation period (108). Patients with comorbid or syndromic conditions are typically admitted for inpatient hospitalization following the procedure. Additional studies examining the postoperative complications across 23 children’s hospitals performing cleft lip repairs suggest that the practice of same day discharge from cleft lip repair is prevalent though discharge practices range from 0 (all patients are admitted for observation) to 60% same day discharge. Those who were discharged same day tended to be older at time of surgery, no preexisting comorbid condition, not have Medicaid, and had surgery at a hospital with higher annual volume of primary cleft lip repair (111). Inpatients were those who had preexisting comorbid conditions, and serious medical complications found in this group included seizures, respiratory failure, and apnea with an estimated 5.5% incidence of significant medical complications (111). Thus, careful patient selection, appropriate home care, and a thorough medical history and physical are crucial for postoperative management in cleft lip patients.

## Outcomes and Quality of Life

Cleft lip repair is a challenging and equally rewarding endeavor, employing an important understanding of esthetics and technical expertise. As previously mentioned, the presentation of the cleft lip can be highly variable and the surgical techniques used to repair the clefts can produce variable results in the hands of different surgeons. There have been numerous papers examining QOL of patients with orofacial clefts that have been well studied, including peer relations, physical health, self-esteem, and psychological stress (112). QOL studies in areas examining social function, social support, family function, school function, however, continue to remain lacking (112). In the most frequently studied QOL measures, results often depended on many different factors and can vary based on the types of questionnaires and variables considered (113, 114). What’s notable, however, is the deficiency of *patient reported outcomes* to measure QOL, as it can play a significant role in identifying QOL aspects that have the greatest need to be enhanced (115). The lack of such an assessment tool was addressed by the Centers for Disease Control in 2006, specifically targeting children with orofacial clefts (116). The difficulty in developing an adequate QOL study for cleft patients lies in the distinct features that must be addressed, including speech, appearance, facial growth, social impact, as well as the relative complexities and importance of each area as the young child matures to an adult. A robust QOL assessment would need to address all these issues (112). The Youth Quality of Life Instrument-Facial Differences (YQOL-FD) was found to be the most effective tool for evaluating patients following cleft surgery (117). However, the YQOL-FD was developed for kids with a broad range of facial deformities and did not specifically address all the unique challenges that may face a clefted child (118). Similarly two voice related measures and two oral health related measures were found to be sufficiently validated for but are not specific toward cleft patients (119). Thus, well developed and well validated patient questionnaires for cleft patients have yet to be developed.

## Conclusion

The congenital cleft lip is a deformity that arises from a genetic or environmental insult during formation of the maxilla and palate in the first trimester of gestation. The etiology of the non-syndromic form is multifactorial and likely involves maternal exposures to teratogens such as tobacco. Cleft lip causes varying degrees of oral sphincter dysfunction, difficulty with speech, and abnormal appearance of the upper lip and nose. The main objectives of surgical repair are to restore normal feeding capacity, speech development, and facial esthetics at an early age before problems arise. Various surgical techniques have been described for definitive cleft lip repair and primary rhinoplasty; the majority are performed between 10 and 12 weeks of age. Bilateral cleft lip presents an anatomical challenge and requires a different surgical approach. Secondary rhinoplasty is frequently required after maturation of the facial skeleton. Adjuncts such as pre-surgical orthopedics are frequently used to optimize surgical outcomes. Long term follow up of the cleft lip patient is mandatory to assess the adequacy of repair and its impact on function and QOL.

## Conflict of Interest Statement

The authors declare that the research was conducted in the absence of any commercial or financial relationships that could be construed as a potential conflict of interest.
